# Acupuncture Treatment for Plantar Fasciitis: A Randomized Controlled Trial with Six Months Follow-Up

**DOI:** 10.1093/ecam/nep186

**Published:** 2011-02-15

**Authors:** Shi Ping Zhang, Tsui-Pik Yip, Qiu-Shi Li

**Affiliations:** ^1^School of Chinese Medicine, Hong Kong Baptist University, Hong Kong, China; ^2^Department of Traditional Chinese Medicine, Southern Medical University, Guangzhou, China

## Abstract

Plantar fasciitis is a common cause of heel pain. It has been suggested that some acupoints have a specific effect on heel pain. The aim of this study was to determine the efficacy and specificity of acupuncture treatment for plantar fasciitis. Subjects were randomly assigned to the treatment group (*n* = 28) or control group (*n* = 25). The treatment group received needling at the acupoint PC 7, which is purported to have a specific effect for heel pain. The control group received needling at the acupoint Hegu (LI 4), which has analgesic properties. Treatment was administered five times a week for 2 weeks, with an identical method of manual needling applied to the two acupoints. The primary outcome measure was morning pain on a 100-point visual analog scale (VAS) at one month post-treatment. Secondary outcome measures included a VAS for activity pain, overall pain rating as well as pressure pain threshold using algometry. Significant differences in reduction in pain scores, favoring the treatment group, were seen at one month for morning pain (22.6 ± 4.0 versus 12.0 ± 3.0, mean ± SEM), overall pain (20.3 ± 3.7 versus 9.5 ± 3.6) and pressure pain threshold (145.5 ± 32.9 versus −15.5 ± 39.4). No serious adverse event was observed in either group. The results indicate that acupuncture can provide pain relief to patient with plantar fasciitis, and that PC 7 is a relatively specific acupoint for heel pain.

## 1. Introduction

Heel pain affects about 10% of the general population and it is commonly caused by plantar fasciitis [1]. Plantar fasciitis is characterized by pain and tenderness centered on the medial tubercle of the calcaneum on weight bearing, especially immediately after rest such as getting out of bed in the morning [2]. The etiology of the disease is unclear. Current conventional treatments include the use of non-steroidal anti-inflammatory drugs and steroid injections [3], but these drug treatments may be associated with serious side-effects [4], and an exploration of alternative treatments is thus warranted.

Acupuncture has been used for many musculoskeletal pain conditions, including heel pain. A thorough literature survey in both Chinese and English electronic databases, including PUBMED, AMED and VIP (a database of Chinese scientific and technological journals), as well as journals of complementary medicine, such as eCAM, up to 2005, identified articles related to acupuncture treatment for heel pain [5]. Only two studies published in Chinese were controlled clinical trials that used acupuncture treatment alone as one of the intervention groups [6, 7]. However, the results from these were controversial, as one of the studies reported that acupuncture treatment was better than local steroid injection [7], whereas the other reported the opposite [6]. In addition, based on current reporting standards [8], both studies lacked scientific rigor and we concluded that further research was necessary to establish the efficacy of acupuncture treatment for heel pain.

A number of mechanisms have been proposed to explain the pain-relieving effect of acupuncture, including central opioid pain inhibition [9], diffuse noxious inhibitory control (DNIC) system [10] and anti-inflammation [11, 12]. Presumably, insertion of a needle at any part of the body may alleviate pain by the mechanisms of opioids or DNIC [9, 13], and the anti-inflammatory action of acupuncture may be generalized across the body. Therefore, a fundamental question often asked is whether needles must be inserted into specific sites to produce the best effect. Despite the obvious importance of this question, only a few clinical studies have succeeded in demonstrating the specificity of an acupoint in pain-related conditions [14, 15] in contrast to a majority of studies showing acupoint specificity in non-pain conditions [16–20].

There are many difficulties in designing clinical trials to demonstrate the specificity of acupoints. For example, acupuncture treatment often involves multiple acupoints, thus it is not possible to isolate the therapeutic effect to a single acupoint, even though there may be a specific effect for a group of acupoints. Furthermore, since the mechanism of acupuncture is not clear, an arbitrary chosen control-acupoint or non-acupoint may in fact produce the same physiological responses as the test-acupoint, and thus it may have the same effectiveness as the test-acupoint. Therefore, in order to examine the specificity of acupoints, it would be desirable to study those treatments using a single acupoint that has a distinct mechanism of action.

For acupuncture treatment of heel pain, two treatment approaches are commonly used: local acupoints or a single distal acupoint [21–24]. The use of a single distal acupoint in treatment of heel pain provides a unique opportunity to study acupoint specificity, even though the mechanism underlying the treatment is still unclear. In this study, we assessed the specificity of the acupoint Daling (PC7) for heel pain, using a nearby acupoint Hegu (LI4) as control. PC7 is used for pain conditions including stomachache, chest or cardiac pain and headache, in additional to heel pain; LI4 has well-known analgesic properties and is commonly used for dental pain, headache and general analgesia [25, 26]. We used identical methods of needling at both PC7 and LI4. The hypothesis being tested was that PC7 had a better effect than LI4 in relieving pain due to plantar fasciitis. If PC7 were indeed more effective than LI4, it would suggest that PC7 not only has a specific effect for heel pain, but also has a mechanism of action different from that observed for LI4.

## 2. Methods

### 2.1. Patient Recruitment

This study was approved by the Committee on the Use of Human and Animal Subjects in Teaching and Research of the Hong Kong Baptist University, and was conducted between 2005 and 2006. Announcements about the study were made in local newspapers and various community centres, and prospective patients were invited to an information session, during which the study was explained. They were informed prior to randomization that the purpose of the study was to evaluate the effect of a specific type of acupuncture for heel pain, and that they would be allocated to either the treatment group or the control group. Initial participant screening involved obtaining a clinical history, examination of the lower limb and recording baseline measurements. These were performed immediately after the information session.

### 2.2. Randomization

Randomization occurred on the day participants returned for their first treatment, somewhere between 5 and 14 days after the initial screening. Using computer generated random numbers, participants were randomized into either the treatment group or the control group. Both the participant and the researcher who obtained the measurements were blinded to the group status of the participant. A coding system was used by the acupuncturist (TPY) to identify the group status to enable the administration of the corresponding treatment.

### 2.3. Inclusion Criteria

Adult participants aged ≥18 years with heel pain for >3 months preceding the study were included. Participants were diagnosed as having plantar fasciitis if the pain was localized to the medial tubercle of the calcaneum, which is the site of the insertion of the plantar fascia and intrinsic muscles of the foot [2]. They were advised to abstain from other forms of treatment during the study period, with informed use of analgesics only if absolutely necessary.

### 2.4. Exclusion Criteria

We excluded participants with a history of fracture or dysfunction of the ankle or knee, or arthritis; with signs of nerve injury; with severe systemic diseases, such as rheumatoid arthritis, diabetes or cardiovascular disorder; who were unlikely to attend all treatment sessions or who were needle phobic, pregnant or breast feeding.

### 2.5. Acupuncture Treatment

The protocol for the acupuncture treatment was based on previous clinical reports [23, 24]. Participants in the treatment group received needling at the acupoint Daling (PC7), which is located on the palmar side of the forearm at the midpoint of the wrist crease [27]. Participants in the control group received needling at the acupoint Hegu (LI4), which is located between the first and second metacarpal bones [28]. LI4 was chosen as the control point because it was close to PC7 and had analgesic properties [25, 26]. Needling was performed at PC7 or LI4 on the contralateral side to the heel pain. If the heel pain was on both sides, bilateral needling of either PC7 or LI4 was performed. Both PC7 and LI4 acupoints were stimulated using sterile acupuncture needles, 15 mm long with a diameter of 0.25 mm (Huatuo, Suzhou Medical Instruments Factory, Suzhou, China). Needles were inserted perpendicularly with the aid of a guide tube, advanced *∼*10 mm deep using slight rotation and thrusting to obtain the Deqi sensation, which was reported by the participants as a dull ache, numbness or heaviness. Needle manipulation was repeated approximately every 5 min to maintain the Deqi sensation, and each treatment session lasted for 30 min. The needling procedures were identical for both the treatment and control groups. Both groups received 10 daily treatment sessions over a 2-week period (usually Monday–Friday). Needlings were performed by a registered Chinese medicine practitioner with 2 years of clinical experience. The practitioner (TPY) had been trained to carry out the treatment procedures as stipulated in the protocol.

### 2.6. Assessments

Initial patient screening involved taking a detailed history and a physical examination of the lower limb. A blinded assistant completed the case report form for the patient at each treatment visit and at the one month follow-up. A questionnaire was posted to participants to follow-up at 3 and 6 months post-treatment. If no reply was received after a week, a telephonic interview was conducted to collect the data.

In order to assess the credibility of the control treatment, the participants' beliefs about the treatment were assessed using a modified Borkovec and Nau scale [28]. At the beginning of the first treatment session and post last treatment session participants were asked to answer four questions on a six-point scale: (i) “How confident do you feel that this treatment can alleviate your complaint?"; (ii) “How confident would you be in recommending this treatment to a friend who suffered from similar complaints?"; (iii) “How logical does this treatment seem to you?" and (iv) “How successful do you think this treatment would be in alleviating other complaints?". Furthermore, participants' perception of acupuncture stimulation was assessed using a questionnaire that had been previously used [29], with modifications so that it could be used at the end of each treatment session. Thus, prior to the administration of the questionnaire participants were informed about the concept of Deqi, a sensation that occurs with needling, which was defined as the feeling of numbness, dull ache, heaviness or a radiating sensation. At the end of each treatment session, they were asked to indicate the intensity (on a 100-point visual analog scale, VAS) and duration of the Deqi sensation. In addition, participants were asked to record their overall perception of needling pain using a VAS.

### 2.7. Outcome Measures

Morning pain is a distinct feature of plantar fasciitis, and thus has been used as the primary outcome measure. Participants were asked to record their perception of heel pain on a 100-point VAS with descriptors at either end (0 no pain; 100 maximal pain). Secondary outcome measures were activity pain (heel pain during activity) and the overall perception of heel pain. Pressure pain threshold was measured using an electronic algometer (SOMEDIC, Sweden) applied by a trained researcher (QSL), prior to each treatment session. Algometry involved a 1 cm^2^ probe placed at the medial tubercle of the calcaneum of the non-painful foot, and at the most painful site on the painful foot (usually the medial tubercle of the calcaneum). The maximum force that could be applied was limited to 1000 kPa for practical reasons. A mean score was obtained from three repeated measurements. Any participant who reported an adverse reaction was noted. Non-study treatments received by participants before, during and after the treatment were also recorded.

### 2.8. Statistical Analysis

For sample size estimation, we presumed that the means and standard deviations of the VAS scores in the control and treatment groups were the same, and were similar to those previously reported [30]. Thus, the respective mean VAS scores and the standard deviations were assumed to be 50 and 20 on a 100-point scale. As a 33%–36% improvement had been considered to be of clinical importance in pain outcome measures [31], we intend to detect a 16.5 unit (33%) difference between the treatment and control groups. With the level of significance set at 0.05 and the power at 80%, a sample size of 50 would be required. Allowing for a dropout rate of 20%, the number of participants required was estimated to be 62.

For data analysis, the SPSS software (version 13) was used. Data were analyzed using Student's *t*-test, Chi-square test, analysis of variance and covariance with Bonforroni correction and regression analysis, as appropriate.

## 3. Results

### 3.1. Participants

**Figure 1:**
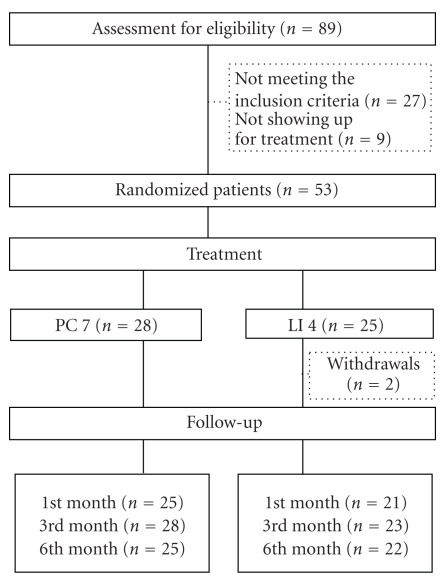
CONSORT chart of the clinical trial process.

Of the 89 subjects who participated in the screening, 62 met the inclusion criteria. Reasons for exclusion were: the onset of heel pain was <3 months (*n* = 2), pain was not located at the heel (*n* = 14), history of foot injury (*n* = 3), rheumatoid arthritis (*n* = 2), unable to attend all treatment sessions (*n* = 3), signs of nerve injury (*n* = 2) and pain in multiple locations of the body with an unknown diagnosis (*n* = 1). After the screening, nine participants failed to return for treatment without giving a reason. Altogether, 53 participants were randomized, with 28 allocated to the treatment group that received needling at PC7, and 25 to the control group that received needling at LI4. Two participants later withdrew from the study in the LI4 group, one after the first treatment session due to intolerance towards the pain associated with the treatment, and the other one after the third treatment session because the heel pain was not improving. In total, 28 participants in the PC7 group and 23 in the LI4 group completed the 10 treatment sessions. Five participants were lost to some of the follow-ups (Figure 1). At the end of the 6-months period, follow-up data were obtained from 25 participants in the PC7 group and 22 in the LI4 group (Figure 1). To determine the difference in treatment outcomes between the two groups, intention-to-treat analysis was carried out with missing data being replaced by the last value carried forward. 

### 3.2. Baseline Characteristics

The general characteristics of the participants are listed in Table 1. It can be seen that most participants were women (71% in PC7 and 76% in LI4 group) and had bilateral heel pain (54% in PC7 and 52% in LI4 group). We asked participants to bring in their X-ray films, if available, and identified the presence of calcaneal spur in 28% of participants in the PC7 group (*n* = 8) and 24% in the LI4 group (*n* = 6). Most participants were receiving some form of treatments prior to entering the trial, but no patient had previously received surgery for heel pain. For both groups of participants, pressure pain thresholds at the painful foot were significantly lower than at the non-painful foot (*P* < .05). The duration of heel pain in the PC7 group ranged from 3 to 216 months, and in the LI4 group ranged from 3 to 144 months, with no statistical significant difference in the mean durations between the two groups (*P* > .05). A negative correlation was found between the duration of heel pain and the morning pain score (*r* = –0.351, *P* = .0132, *n* = 53), suggesting that morning pain decreased as the duration of heel pain increased. Overall, no significant difference existed in the baseline data between the two groups. During the intervention period, no subject reported the use of other treatments. 

### 3.3. Changes in Outcome Measures

**Figure 2:**
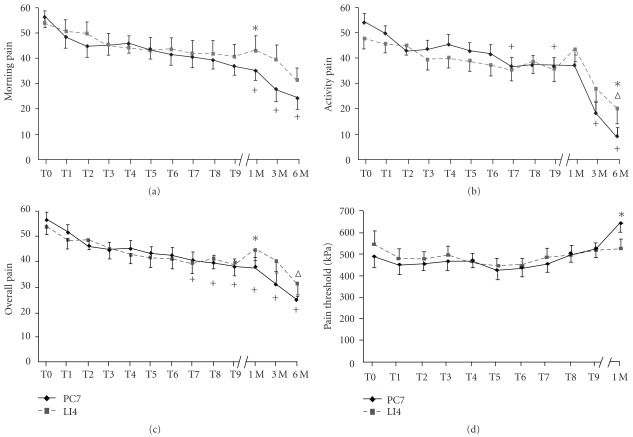
(a)–(d) Line graphs showing the effects of acupuncture treatment at two different acupoints. Patients were randomized into the treatment group (*n* = 28), receiving acupuncture at acupoint PC7 (diamonds), and the control group (*n* = 25), receiving acupuncture at acupoint LI4 (squares). Values are mean ± SEM taken at different time points. T0, just prior to the first treatment; T1, after the first treatment and just prior to the second treatment; T9, after the ninth treatment just prior to the tenth treatment; 1 M, 1 month post-treatment; and so forth. Crosses and empty triangles indicate statistical significant differences compared with T0 in the PC7 group and the LI4 group, respectively (*P* < .05; one-way ANOVA with Bonferroni *post hoc* test). Stars indicate statistical significant differences between the PC7 and LI4 groups at a given time point using a multivariate general linear model, with baseline values as covariate (*P* < .05, with Bonferroni correction).

For the PC7 group, compared with the baseline value, a significant improvement in morning pain was observed from one month post-treatment to the 6-month period (*P* < .001, ANOVA with Bonferroni *post-hoc* test). For the secondary outcome measures, gradual improvements from baseline values were seen in activity pain and overall pain following the seventh treatment session and continuing up to 6 months (*P* < .05), except that changes were not significant at the eighth treatment session and at one month post-treatment (*P* > .05). The changes in pressure pain threshold were not statistically significant compared with the baseline. A negative correlation between the duration of heel pain and the effect of the PC7 treatment in morning pain was found (*r* = –0.399, *P* = .039, *n* = 28), suggesting that the treatment was more effective for participants who reported a shorter duration of heel pain. In contrast, the LI4 group showed no improvement in morning pain. The only significant improvements for this group were activity pain and overall pain at 6 months (*P* < .05, ANOVA with Bonferroni *post-hoc* test). Furthermore there appeared to be a rebound effect at one month post-treatment in the control group (Figure 2).

**Figure 3:**
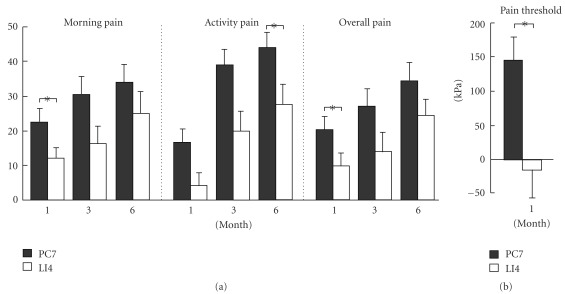
Histograms showing mean changes of morning pain, activity pain, overall pain and algometric pain threshold from the baseline in the specific acupoint group (PC7, *n* = 28) and the non-specific acupoint group (LI4, *n* = 25). Number of feet in algometric measurement: PC7, *n* = 43; LI4, *n* = 38. *P < .05.

Of primary interest is whether there was any difference in the improvements between the PC7 and LI4 groups. Using a multivariate general linear model in the SPSS software with baseline values as covariate and applying Bonferroni correction, significant differences were detected at one month for morning pain (*P* = .044), overall pain (*P* = .049) and pressure pain threshold (*P* = .007), favoring the PC7 group. Difference in activity pain was also observed at 6 months (*P* = .048; Figure 2). The differences in improvements of outcome measures between the two groups are also summarized in Figure 3 to illustrate the effect size of treatment. 

### 3.4. Perception of Acupuncture Stimulation and Credibility Rating

**Figure 4:**
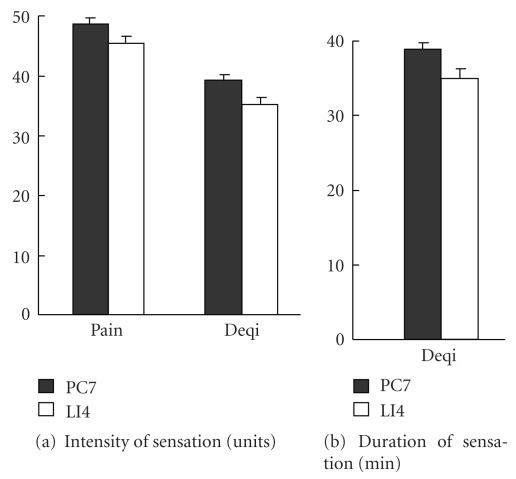
Assessment of perception of acupuncture stimulation. (a) Intensity of pain or Deqi sensation. (b) duration of Deqi sensation. No difference was found between the two groups.

Participants' perception of Deqi sensation and pain associated with acupuncture treatment are summarized in Figure 4. There was no difference in the duration and intensity of Deqi sensation or in the intensity of pain between the two groups. Thus, the perception of the acupuncture stimulation appeared to be similar for the two groups.

**Figure 5:**
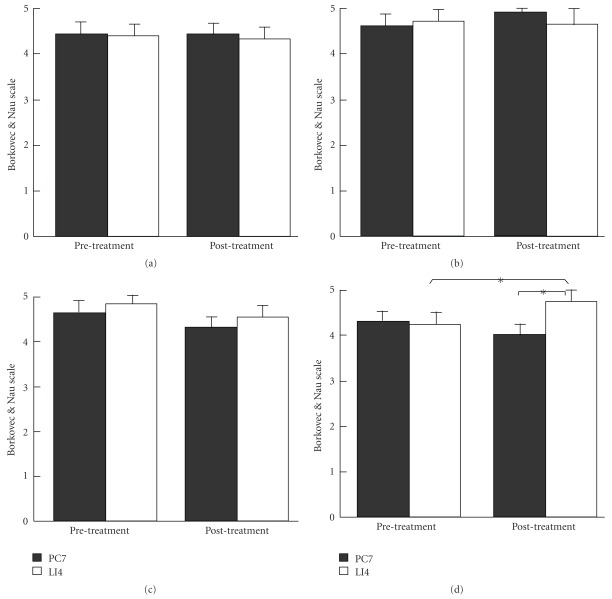
Credibility assessment of acupuncture treatments using a six point Borkovec and Nau scale. (a) How confident do you feel that this treatment can alleviate your complaint? (b) How confident would you be in recommending this treatment to a friend who suffered from similar complaints? (c) How logical does this treatment seem to you? (d) How successful do you think this treatment would be in alleviating other complaints? (*P < .05; PC7: *n* = 28, LI4: *n* = 25).

The results of Borkovec and Nau scale used to assess credibility are shown in Figure 5. It can be seen that there was no difference in the score of each question before the treatment sessions between the two groups. For the PC7 group, there was no difference in the scores before and after the treatment sessions. However, for the LI4 group, the score for the question of “How successful do you think this treatment would be in alleviating other complaints" was increased after the treatment sessions, which was also significantly higher than that of the PC7 group. In other words, participants in the LI4 group became more confident in acupuncture for alleviating other complaints towards the end of the treatment course. When the results of perception of acupuncture stimulation and credibility rating taken together, it was apparent that needling acupoint LI4 had been perceived by the participants as a highly credible form of treatment in the current experimental setting.

### 3.5. Adverse Reaction

No severe adverse reaction was seen in either group. One patient withdrew from the treatment in the LI4 group because the treatment was too painful. A total of 8 participants (28.6%) in the PC7 group and 10 (40%) in the LI4 group reported mild adverse reactions other than pain. Specifically, mild local edema around the area of needling was the second most common reaction next to pain (PC7: *n* = 2; LI4: *n* = 7). Bruising was the third most common adverse effect (PC7: *n* = 4; LI4: *n* = 5). One patient in the PC7 group reported a distressed sensation in the chest on three occasions.

## 4. Discussion

This is the first study investigating the efficacy of a single acupoint for plantar fasciitis, and one of few studies that examines the specificity of a single acupoint for chronic pain. We found statistical differences in the primary and secondary outcome measures between the PC7 group and the LI4 group primarily at the one month post-treatment period. Our findings suggest that relative to LI4, acupoint PC7 has specific effect on heel pain due to plantar fasciitis.

In acupuncture treatment, four major factors may contribute to the improvement of symptoms: the spontaneous resolution of the condition, the placebo or psychological effect that is similar to that observed in participants receiving placebo medication, a general or non-specific physiological reaction to needling irrespective of site, and finally, a specific effect due to needling at an appropriate location [32]. Depending on the research question being addressed, different control methods have previously been used. For example, to answer the question of whether acupuncture treatment is better than no acupuncture treatment, non-stimulating techniques, including “placebo" needle or non-insertion of needle, sham laser and sham transcutaneous electrical nerve stimulation (TENS), have been used as controls. To determine whether needling of the intended sites is better than needling of irrelevant sites, inserting the needles to “inappropriate" points with intensity of stimulation similar to the real treatment would be the choice of control. This method of control has been used to study several pain conditions, including low back pain [15], firomyalgia [33] and post-operative pain [14], but contradicting results have been found with regards to the importance of needling location. Furthermore, recent large-scale clinical trials found that minimal acupuncture (superficial needling) at “inappropriate" points had the same efficacy as acupuncture at intended points for headache [34], low back pain [35] and migraine [36]. However, another study in patients with osteoarthritis of the knee found pain and joint function were improved more with acupuncture at intended points than with minimal acupuncture at “inappropriate" points or no acupuncture after 8 weeks of treatment [37]. Using an “inappropriate" point with similar intensity of stimulation as control, the present study found that the acupoint PC7 was more effective than the “inappropriate" acupoint LI4 for heel pain. There are a few major differences between the present study and those studies that fail to find acupoint specificity. For example, we compared one distal acupoint with another “inappropriate" distal acupoint, whereas previous studies compared multiple intended acupoints with multiple “inappropriate" acupoints, some of which were located in the proximity of the diseased region. Since the local “inappropriate" acupoints may produce similar neurophysiological responses as the nearby intended acupoints by means of segmental reflex response, they may have a similar efficacy as the appropriate points. Furthermore, heel pain of plantar fasciitis is a homogenous pathological condition characterized by local inflammation. Although we used pain perception as outcome measures, the slow onset and long-lasting effect of pain reduction suggest that the improvements observed in the current study were more likely due to resolution of the underlying inflammation or the anti-inflammatory effect of acupuncture, rather than inhibition of pain processing or an immediate analgesic effect. Taken together, it is conceivable that different mechanisms are involved in acupuncture treatment of different pain conditions, and the effect observed for PC7 in treatment of heel pain is related to an anti-inflammatory action, rather than an analgesic action, which has been observed previously for LI4 [25, 26].

A drawback of this study is that it does not include a second control arm, in which participants do not receive any active acupuncture treatment. Thus we were not able to assess the efficacy of the intended acupuncture treatment compared with placebo. In the current study, the differences between the treatment and control groups in morning pain, activity pain and overall pain were around 10–12 points on a 100-point VAS scale (Figure 3), or about 20% of the 50–60 baseline pain values. These differences do not reach the 33% threshold pre-defined as having clinical significance at the beginning of this study. However, the control group also showed gradual improvements from the baseline, although most of these improvements were not statistically significant. The gradual improvement may be related to the effects of non-specific physiological responses evoked from needling LI4, or due to spontaneous resolution of the condition. If we determined the effect size of the treatment based on the differences between the PC7 and LI4 groups, the possible non-specific physiological responses evoked from LI4 would lead to underestimation of the efficacy of PC7. A further study should be carried out to determine the efficacy of PC7 using a parallel non-treatment control group.

Nevertheless, a positive conclusion can be made from this study regarding the efficacy of acupuncture if one views the results from the patient's prospective. That is, the baseline morning pain was reduced by 22.6 points from a base line of 55.3 points at one month follow-up in the PC7 group (Figure 3). This represents a 40% reduction of pain from the baseline, which would be of significance to the patient [31]. Therefore, though the study failed to demonstrate any clinically significant difference between the two groups, our findings do suggest that participants receiving treatment at acupoint PC7 would experience clinically relevant pain reduction from one month onwards, although such relief may be due to a combination of factors, such as the specific effect of PC7, non-specific physiological and psychological responses and the spontaneous resolution of the disease. In contrast, in the LI4 group such clinical relevant pain reduction could only be found 6 months after the treatment.

In studies of steroid treatments for plantar fasciitis, Gudemen showed a difference in the Merryland Foot Score favoring dexamethasone at 2-3 weeks but not at one month, when comparing the effects of an inotophoretic application of dexamethasone and saline [38]. In comparing steroid injection with saline control injection, Crawdford reported that the weighted mean difference in heel pain was 1.94 on the 10 cm VAS score at one month but no significant difference thereafter [3]. Our study also found that most of the significant differences in outcome measures between the treatment and control groups fell on the one month follow-up, suggesting that both steroid injection and acupuncture treatment share a similar time course of maximal effectiveness. It is not possible to compare the efficacies of the current specific acupuncture treatment with that of the steroid injection, as the controls and other aspects of the two trials are quite different. It would be worthwhile, however, in future studies to compare the efficacies of acupuncture treatment and steroid injection.

Although most of the statistical significant differences between the two groups were found at one month post-treatment, statistically significant improvements from the baseline were seen in the treatment group after the seventh treatment session for activity pain and morning pain. This time course of treatment effect is in agreement with previous clinical reports showing that pain relief from acupuncture can be seen as early as 1-2 treatment sessions [39, 40]. On the other hand, there were few statistical differences between the two groups at the 3 and 6 months post-treatment periods. This may be due to the small sample size of the current study, the self-limiting nature of the disease or participants receiving other modalities of treatment during the follow-up period.

**Figure 6:**
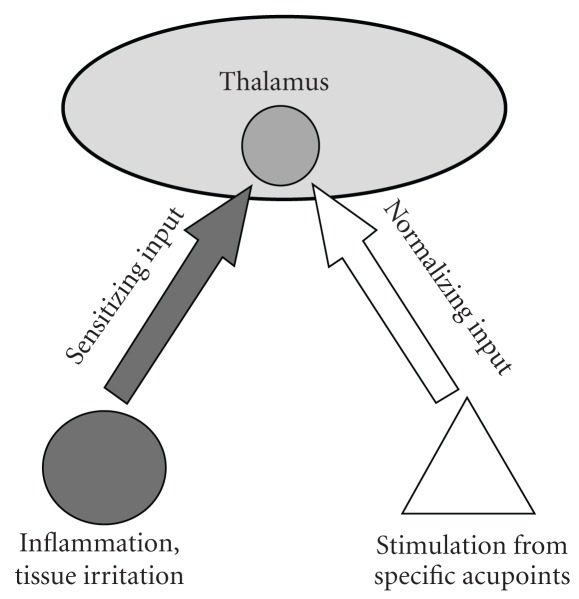
A hypothetical diagram illustrating the possible mechanism of acupuncture treatment for plantar fasciitis. Repeated stimulation from inflammation and tissue irritation of the heel will sensitize neurons in the thalamus and habituate them to a state of hyperexcitability, leading to a state of chronic pain. Repeated stimulation from specific acupoints will send input to the same thalamic focus and normalize the excitability of hyperexcitable neurons.

Although traditional acupuncture theory includes a point selection principle of using points of the upper extremity to treat disease of the lower extremity, and vice versa, no specific acupoint has been proposed for treatment of heel pain from this theory. The underlying mechanism for the specific effect observed is not at all clear. The slow onset and gradual time course of the effects appear to rule out any important contribution of the endogenous opioid system or the DNIC mechanism, as both mechanisms are fast but short acting [9, 41]. In the Chinese literature there are many examples of using individual distal acupoints for pain or inflammation, such as the use of Yaotongdian (EX-UE7) at the hand for acute low back pain, the use of Er Bai (EX-UE2) at the lower arm for hemorrhoids and the use of Quchi (LI 11) for refractory uraemic pruritus [17, 27]. Our results support the purported theory that there exist specific acupoints for the corresponding pain or inflammatory conditions. It has been proposed that prolonged or repeated stimulation habituates neurons in the thalamus to a state of hyperexcitability, leading to a state of chronic pain [42], and stimulation of specific acupoints may adjust the excitability of hyperexcitable neurons in the thalamic focus (Figure 6). In this regard, it is interesting to note that the center of the wrist crease on the palmar side, where PC7 is located, is an anatomical mirror site of the heel. The exact reason for the possible corresponding effect is worthy of further investigation. Functional brain imaging studies may provide insight into mechanisms of the neural network underlying the specificity of acupoints [43]. 

In conclusion, this study demonstrates that acupoint PC7 has a specific effect for treatment of plantar fasciitis, and that the method of acupuncture treatment is both simple and safe. Further studies comparing acupuncture treatment with an inert placebo and conventional treatment as parallel arms are recommended to further elucidate the efficacy of acupuncture treatment for heel pain.

## Funding

Partially supported by a Faculty Research Grant from the Hong Kong Baptist University.

## Figures and Tables

**Table 1:** Demographic and baseline data.

Characteristic	Mean ± SEM; *n* (%)	
	PC7 (*n* = 28)	LI4 (*n* = 25)
Age (years)	47.0 ± 2.2	50.0 ± 2.0
Height (cm)	161.4 ± 1.8	159.2 ± 2.3
Weight (kg)	64.8 ± 2.3	66.7 ± 2.6
Sex		
Male	8 (28.6)	6 (24)
Female	20 (71.4)	19 (76)
Affected side		
Left	7 (25)	7 (28)
Right	6 (21.4)	5 (20)
Bilateral	15 (53.6)	13 (52)
Duration of heel pain (months)	44.9 ± 8.8	22.9 ± 8.8
Weight bearing time (h/day)	5.8 ± 0.7	4.6 ± 0.6
Calcaneal spur shown in X-ray	8 (28.6)	6 (24)
Not receiving any treatment	2 (7.1)	4 (16)
Previous treatments	26 (92.9)	21 (84)
Western medicine	13	14
Local injections	4	5
Surgery	0	0
Chinese medicine	10	7
Acupuncture	12	4
Physiotherapy	11	7
Foot pad	18	13
Cream or tape	15	0
Others	1	0
Morning pain ^(a)^	55.3 ± 4.6	53.2 ± 4.7
Activity pain ^(a)^	54.5 ± 3.1	48.3 ± 4.1
Overall pain ^(a)^	58.1 ± 2.6	55.0 ± 3.4
Pain threshold (kPa)		
Normal foot	835.2 ± 34.6	873.9 ± 35.3
Affected foot	466.9 ± 34.0	522.7 ± 48.4

^(a)^0–100 point scale.
